# Culturally Competent Informed-Consent Process to Evaluate a Social Policy for Older Persons With Low Literacy: The Mexican Case

**DOI:** 10.1177/2158244016665886

**Published:** 2016-08-22

**Authors:** Emma Aguila, Beverly A. Weidmer, Alfonso Rivera Illingworth, Homero Martinez

**Affiliations:** 1 University of Southern California, Los Angeles, USA; 2 RAND, Santa Monica, CA, USA

**Keywords:** demographics, diversity and multiculturalism, ethics, policies, social science

## Abstract

The informed-consent process seeks to provide complete information to participants about a research project and to protect personal information they may disclose. In this article, we present an informed-consent process that we piloted and improved to obtain consent from older adults in Yucatan, Mexico. Respondents had limited fluency in Spanish, spoke the local Mayan language, and had some physical limitations due to their age. We describe how we adapted the informed-consent process to comply with U.S. and Mexican regulations, while simplifying the forms and providing them in Spanish and Mayan. We present the challenges and lessons learned when dealing with low-literacy older populations, some with diminished autonomy, in a bilingual context and a binational approach to the legal framework.

## Introduction

Ethical and legal considerations require that human subjects who participate in research do so under informed consent. This requires them to receive a detailed, complete, and comprehensible description about research objectives, an explanation why they are being asked to participate, and assurance that their participation is voluntary and that declining to participate will not have negative consequences. Processes for gaining informed consent are reviewed by an Institutional Review Board (IRB), with attention to the level of risk, characteristics of the target group, and cultural practices of the population (Faden & Beauchamp, 1986; Gostin, 1991).

Gaining informed consent may be complicated for populations with low levels of literacy, limited language fluency, or different cultures than researchers (Kripalani, Bengtzen, Henderson, & Jacobson, 2008). It may be even more difficult when working with the elderly, persons in rural areas, or places where a required signature on a document may cause anxiety or suspicion about the intentions of those requesting it (Wendler, 2004).

The importance of informed consent dates back to the 1947 Nuremberg Code, drafted as evidence emerged of abuse in human-research trials during the Second World War, and then again in the 1964 Helsinki Declaration. Informed consent did not become routine practice until the 1960s, and was not enforced until Pappworth (1967) in the United Kingdom and Beecher (1966) in the United States published details of prevalent unethical exploitation of “marginalized minority groups” in experiments “without their knowledge or consent.” Informed-consent guidelines were first developed for medical and clinical research, with such guidelines being more standardized than those for social-science research (Beauchamp & Childress, 2012).

The issue of attaining informed consent among marginalized populations deserves special attention for several reasons (Beecher, 1966; Pappworth, 1967). Marginalized populations have been systematically excluded in clinical research because of imperfect and culturally insensitive informed-consent procedures, researcher bias (e.g., “belief that minorities will not agree to participate or adhere to protocols,” as discussed in Barata, Gucciardi, Ahmad, & Stewart, 2006, p. 480), and researcher perception of costs (such as those to translate informed-consent materials, as discussed in Swanson & Ward, 1995).

Such considerations become more complicated in foreign countries, where specific requirements and processes for obtaining informed consent must conform both to the regulations of where the research is conducted and to those of the foreign partner (U.S. Department of Health and Human Services, 2009). Researchers doing international work may need creative approaches to comply with requirements from domestic and foreign IRBs.

In this article, we review efforts to gain informed consent among participants in an income-supplement program in Yucatan, Mexico, in a way that was acceptable to both the host country for the research and the home country of the researchers. As part of its social policies to improve the quality of life for older adults, the Yucatan state government collaborated with researchers from the RAND Corporation, the a (USC), and other U.S. and U.K. institutions to design, implement, and evaluate an income-supplement program for persons aged 70 and older (Aguila, Kapteyn, Robles, Vargas, & Weidmer, 2014).

The state of Yucatan occupies the northern part of the Yucatan peninsula in Southeastern Mexico. Nearly one third (32.6%) of its population lives in rural areas (Instituto Nacional de Estadística y Geografía [INEGI], 2010), and nearly half (45.7%) lives in poverty (Consejo Nacional de Evaluación de la Política de Desarrollo Social [CONEVAL], 2012). Poverty is higher in Mexico among those who speak an indigenous language (76%) than it is among those who do not (43%); Yucatan is the state with the highest proportion (51%) of inhabitants who speak an indigenous language (CONEVAL, 2014).

The program included a detailed survey of selected household members on demographic characteristics, income and assets, employment history, expenditures on durable and non-durable goods, family transfers, health and physical status, access to and use of health care, medication use, social networks, social support and caregiving responsibilities, dietary intake, and food security. For eligible individuals, it also included anthropometric and selected biomarker measurements (Aguila et al., 2014).

Many individuals had limited literacy, and most spoke the native indigenous language, Mayan, with only limited fluency in the national language, Spanish. Many of these persons also had limited autonomy given their age. In developing informed-consent processes for them, we paid particular attention to developing a culturally appropriate process that conformed to both U.S. and Mexican norms and regulations for conducting social-science research involving human subjects.

This study expands the scant information previously available on gaining informed consent among older individuals outside of community centers or nursing homes. Taub, Kline, and Baker (1981) found that special methods may be needed to present informed-consent processes to elderly persons with limited vocabulary and low levels of education. Previous research noted that older persons in nursing homes have difficulties in providing informed consent, given their levels of mental competence (see, for example, Annas & Glantz, 1986; Cohen-Mansfield, Kerin, Pawlson, Lipson, & Holdridge, 1988).

## Method

### An Overview of the Informed-Consent Process

Human subjects who are invited as potential participants on research projects should be considered autonomous individuals, capable of deciding their own acts after deliberating whether to participate in a project (National Commission for the Protection of Human Subjects of Biomedical and Behavioral Research [NCPHS], 1978). As such, they should be provided with all pertinent information about the project, including potential risks and benefits, as well as assurance that their decision will not influence any benefit, such as the right to health care or government benefits, to which they may be entitled (Gostin, 1991).

Researchers should ensure that potential participants clearly understand participation is voluntary, free of coercion, and can be terminated any time without any undesirable consequence to their rights (NCPHS, 1978). Researchers must also take proper measures to protect the confidentiality of the information collected, including the participant's identity and any sensitive information that may place the individual at risk if it were to be disclosed (Organization of American States, 2012; Penslar, 1993).

The informed-consent process and efforts to protect privacy of participants begin before contacting the research participants, continue throughout project implementation, and extend to the use of data and dissemination of results. When explaining research methods to a potential participant, the research team must use language and concepts that the participant can understand (NCPHS, 1978; Penslar, 1993). Participants have the right to resolve their doubts and ask questions about project aims and objectives, possible risks or expected benefits, and what will be done to address psychological, physical, or moral harm that may occur as a result of project participation (NCPHS, 1978; Penslar, 1993).

Once all these issues have been properly addressed, potential participants may give their informed consent to take part in the project. Documentation of this informed consent is usually collected on paper using a digital or hand-written signature (CONEVAL, 2012). In some cases, participants may waive written consent but provide oral consent (Klitzman, 2013). Beyond ethical and moral obligations, informed consent has legal consequences for participants and the institution responsible for the research (Klitzman, 2013; Penslar, 1993).

### U.S. and RAND's Protection for Human Subjects Participating in Research

In the United States, organizations receiving federal research funds must have an IRB to ensure compliance with federal regulations regarding human-subject protection (Bankert & Amdur, 2006). Such IRBs must register with the Department of Health and Human Services. To register, an IRB must have a Federal Wide Assurance (FWA) for the Protection of Human Subjects, which must be renewed every 3 years. The FWA is the only means accepted by the Office for Human Research Protections to assure that the institution will comply with federal requirements (U.S. Department of Health and Human Services, 2009).

In line with these policies, RAND's IRB, known as the Human Subjects Protection Committee (HSPC), is responsible for reviewing every research project undertaken by the institution, regardless of the source of funding and project location. Every RAND researcher must submit a research protocol for HSPC approval before data collection begins, usually after funding has been assigned a “fundable” score (in the case of National Institutes of Health [NIH] projects), committed or received. Every principal investigator (PI) should be trained in the protection of human subjects and be cognizant of HSPC procedures before submitting a project for review (NCPHS, 1978).

PIs are responsible for preparing the required materials for submission, responding to any queries raised by HSPC reviewers, and complying with HSPC recommendations and regulations before engaging in any activity that involves human subjects. They are also responsible for reporting any event that departs from the expected course of the project, as well as any event that may harm participants in the research, regardless of whether this is related to research procedures (NCPHS, 1978). Likewise, every PI is responsible for submitting any changes to the research protocol initially approved, whether it is an amendment to project procedures, a request for approval of any new component to the project (e.g., a new survey), or a progress report, as long as the project is kept “active” by HSPC. After the project is closed, the PI is responsible for safeguarding signed informed-consent forms, as well as for protecting participant confidentiality in presentations or publications of results.

Such responsibilities extend to all staff considered to be RAND's “agents,” including project members hired by RAND directly or indirectly (i.e., through an in-country sub-contractor, such as an academic or a public institution). This requires that local staff involved in any RAND project receive adequate training in human-subject protection and adhere to RAND policies and procedures for protecting the research participants (NCPHS, 1978).

### Requirements for Protecting Human Subjects Participating in Research in Mexico

Government-regulated procedures for protecting human subjects in Mexico have a shorter history than they have in the United States. Although the General Health Law, which regulates human-based research, established informed consent as a legal requirement in the mid-1980s (Cámara de Diputados del Congreso de la Unión, 1984), not until 1992 did the Ministry of Health establish the National Commission of Bioethics (NCB). Following a presidential agreement in 2000, the NCB became a permanent body with technical and operational autonomy (Diario Oficial de la Federación, 2005).

The NCB is responsible for establishing and promoting health policies related to bioethics, encouraging the creation of Bioethics Commissions for each state of Mexico, establishing guidelines and criteria for the activities of these committees, and promoting bioethics education among health care institutions (Comisión Nacional de Bioética [NCB], n.d.). The mission of NCB to promote norms and guidelines for research ethics committees includes the informed-consent process for social and biomedical research (NCB, 2005).

Every public, private, or social institution that operates under the National Health System and conducts research involving human subjects should have a Local Research Ethics Committee (LREC), equivalent to an IRB in the United States. LRECs are responsible for reviewing and applying research ethics to all types of research, but typically such reviews are reserved for projects that have human subjects (Valdez-Martinez, Turnbull, Garduño-Espinosa, & Porter, 2006). Institutions that conduct research involving human subjects are mandated by law to submit their research protocol to a LREC.

Social-science and biomedical research conducted in Mexico historically has had little regulation, with many research projects routinely failing to use an informed-consent protocol (Garcia, 2009; Lamas et al., 2010; Outomuro, 2008). Although the commission promotes LRECs as part of its capacity-building mandate, their establishment is left to universities, research centers, or other research entities (Comisión de Salud Fronteriza México-Estados Unidos, 2010).

Complying with the law can take time, so many Mexican universities and research centers have no LRECs. This is particularly evident in institutions that do not regularly carry out research. State-level ministries of health are not required to (and often do not) have LRECs. At the time of our research, Yucatan was not among the 20 states (of 32) that had formally established a LREC.

Researchers collaborating with these institutions may find it necessary to find another institution, such as a local university with an LREC, to review their study. There are no guidelines for such review, and there is often a disconnect between the reviewing institution's LREC and the goals and needs of the proposed research. As a result, the review process may be long and tedious, often discouraging researchers from going through it (Comisión de Salud Fronteriza México-Estados Unidos, 2010). This can make binational research difficult given U.S. requirements for IRB approval.

According to the NCB (2005), informed consent involves a social process that should provide information about the research in an understandable way to the respondent, allow the researcher to verify that the respondent understands what participation involves, and provide the opportunity for the respondent to ask questions and have them answered. The process must also allow the respondent to decline participation without being subject to intimidation, coercion, or being unduly influenced by incentives that could be viewed as coercive (Aguilera-Guzmán, Mondragón Barrios, Icaza, & Elena, 2008).

In practice, informed-consent processes in Mexico focus more on the completion of the consent form as a legal requirement than on the process of actually acquiring effective “informed” consent. A shortage in adequate training, guidance, and supervision of LRECs results in reviews that focus on rules, regulations, and improving research methods and analyses rather than on protecting the rights and well-being of participants (Angeles-Llerenas, Wirtz, & Lara-Alvarez, 2009; Valdez-Martinez et al., 2006).

### Developing Informed-Consent Methods for Our Project Population

Requirements for our project included asking for informed consent from participants at each stage of the survey. Specifically, we asked respondents for their consent to take part in the general survey (oral), allow the research team to review administrative and health records (written), and collect anthropometric and biomarker measurements (written), while assuring informants that we would protect individual confidentiality. Developing consent forms that could easily explain the aim of the project to the participants posed a challenge, given their age, language barriers, low levels of schooling, and cultural characteristics (Gostin, 1991). We sought to respect the autonomy of the individuals, especially given that some participants had physical constraints. The project also had to comply with both U.S. and Mexican regulations regarding research with human subjects.

To develop the informed-consent documents, we first reviewed forms previously used in other projects, particularly those similar in scope and complexity and that had used written rather than oral consent. We developed an initial version of the consent forms in English for RAND HSPC review, then translated them into Spanish.

Before moving into the field, all field staff underwent a 2-week training course on study procedures and selected aspects of human-subject protection. This course included practical interview topics, such as how interviewers should introduce themselves and the project to potential participants, as well as administrative procedures, including data-safeguarding plans and handling of potential adverse events. Training included theory (lectures) and practice (group sessions to enact interviewing procedures). To promote a culturally sensitive interaction between respondents and interviewers, a local anthropologist gave a lecture to interviewers about Mayan culture and the role of the elderly in society. Training was followed by the pilot field-test phase, in which interviewers visited homes to collect written informed consent and test the data-collection forms.

A first pilot test was conducted at a nearby site, in a locality called Progreso, away from the area where the project was to be conducted. This pilot test involved approximately 200 Spanish-speaking participants and included the application of the informed-consent forms as well as the full battery of planned individual- and household-level data-collection procedures. We requested written informed consent for the survey, for access to administrative or health records from any public program in which the subject had taken part, and for anthropometric and biomarker measurements. Participants who signed the informed-consent form were given a copy for future reference.

We followed the same procedures in each pilot test to identify issues and challenges of the survey application. The PI, survey director, local project director, and field coordinator accompanied the field staff during these pilot tests to observe directly the informed-consent process. Following each field-test, all project staff held two extensive debriefing sessions to identify salient issues and major challenges.

The first debriefing session was held the last day of each pilot test between interviewers and supervisor. Interviewers reported their findings to their supervisor who made notes and merged these comments with his or her own observations during the pilot test. The PI, survey director, local project director, and field coordinator also attended and made notes in these debriefings. The first debriefing sessions lasted on average 2 hr each. At the end of this session, supervisors summarized the main issues and challenges for the field coordinator. The field coordinator, in turn, summarized, by topic, main issues, and challenges identified in the debriefing sessions. A report for discussion was then presented to the PI, survey director, local project director, and a local researcher expert on primary data collection. This subsequent meeting sought potential solutions to the challenges faced in the pilot test and to highlight items requiring further information.

The second debriefing session summarized the issues and challenges faced by all interviewers and supervisors, and discussed potential solutions with all field staff. The second debriefing session lasted on average between 3 and 4 hr, and included 35 staff members: all five teams (each comprising five interviewers and one supervisor), the field coordinator, local project director, survey director, a local expert on primary data collection, and the PI. During the first 30 to 40 min of the second debriefing session, the field coordinator presented the main issues raised in the first debriefing session. In the next 60 min, participants provided feedback of additional items not considered in the summary or provided deeper insights about a specific item. In the next 90 min, participants had a brainstorming session on solutions to the issues presented. The survey director led the debriefings and organized the discussion. Supervisors, the local project director, the field coordinator, the local expert on primary data collection, and the PI all made notes during the second debriefing sessions. After the second debriefing, the field coordinator revised the summary of issues and challenges with suggested solutions, using her notes and the debriefing notes of supervisors, the local project director, the local expert on primary data collection, and the PI. The field coordinator provided this report to the survey director and local expert on primary data collection, who then made a final decision on the feasibility of changes to the informed-consent protocol and who prepared a report for the RAND IRB. The revised forms were drafted in Spanish, and the local expert on primary data collection helped simplify them (Aguila, Cervera, Martinez, & Weidmer, 2015). Revised consent forms and reports of the field-tests were then translated into English, to be submitted as an amendment to the report for RAND HSPC.

After the debriefings of the first pilot test in Progreso, we realized that the informed-consent forms needed to be translated to Mayan for respondents who could not fully understand the process in Spanish. Bilingual (Spanish–Mayan) staff reviewed all forms prepared by professional Mayan translators for content and accuracy. We then conducted a second pilot test in a nearby town, Teabo, using the Mayan-language versions of the informed-consent and survey instruments. The translation of the instruments was a major enterprise that increased project costs but was necessary to conduct research with the indigenous elderly.

The informed-consent forms were provided in both Spanish and Mayan to all project participants, who were allowed to keep a copy for future reference. All interviews and procedures were conducted in a place that provided comfort and privacy for respondents, whether in their own home or, if respondents so chose, in a place such as a school, church, or government office. Interviewers made sure respondents understood that they could refuse to answer any question, participate in one component of the project but not another, or withdraw from the project at any point, without giving up any state-government benefit to which they were entitled.

## Results

During the debriefing sessions held by project and field staff after the first pilot test, it became obvious that many participants were intimidated by the consent form and process. There were three issues causing problems.

First, many respondents, particularly those with low levels of education, were not able to understand the content and language of the forms. The length of the forms and the time they took to discuss (as much as 30 min) proved too tedious for many potential participants. Many became tired, and lost attention, interest, or willingness to participate. Many of our interviewers were also unfamiliar with informed-consent processes. While most held an undergraduate degree, and 30% had participated in previous data-collection projects, none were familiar with the human-subject protection process before our training.

Second, many respondents were illiterate or had poor vision; hence, they were unable to read any of the forms. As described by an interviewer, one male participant who lived alone in the city of Progreso “gladly agreed to participate” in the project. However, referring to the consent forms, he said, “I can participate but I cannot sign them because I cannot see well and I do not have glasses; therefore, I prefer not to sign.” Others who could not read the forms were afraid to sign something they were unable to read, for fear of being tricked into something that they could not independently verify. In some instances, having a family member or a field interviewer read the form out loud helped. Still, the written nature of the documents posed an obstacle for a number of participants.

Third, participants were intimidated by the consent process and, particularly, signing the consent forms. The lack of knowledge and understanding of consent forms caused many respondents to fear that someone would somehow take advantage of them. In some cases, respondents refused to sign the consent forms though they indicated willingness to participate in the study. The requirement to sign document made them suspicious of the intentions of the field staff.

These suspicions could relate to fears of being duped by, or associated with, criminals. The words of a female participant, as recalled by an interviewer, provide an example of these fears:

I will not sign any documents because I'm afraid that you can use my information for some illegal purpose, as I had heard on the news about fraudulent calls being made to old persons in which strangers said that they have personal information of a family member and they used them for criminal purposes if they did not give money.

She also mentioned being wary due to cases she had heard in which criminals used others’ names and signatures to steal money from bank accounts. The participant had no problem understanding the project or forms. She simply refused to provide her signature due to fears of theft and fraud.

In another case of refusal, when asked for her signature, a female participant said, “No, I'm afraid you guys might take away my property or belongings.” After more conversation and explanation by the interviewer, she still asserted, “I do not know, because you could change everything and make this like a distraction to take away the small valued things that I have left.”

Respondents also feared that “the government” would take advantage of them. For example, a female participant who refused to sign the consent form said, “The government only wants our signatures to get more money, so the people in the government can continue robbing our money.” Another female participant said,

I detest everything related to the government; if I accepted to collaborate with you, it's because I care about people like you who are just doing their job to support your families, but I'm not going to sign any official document to the government. I do not want to be associated with the government in any way.

Family members sometimes reinforced such fears, actively discouraging or preventing their elder relative from signing the form. They feared that a signature would compromise their property or possessions, or waive some privileges related to government-sponsored welfare programs. In the words of one participant's wife, who supported her husband's decision to participate without signing the consent form, “We found the study very interesting and we're very grateful for having been taken into consideration. However, we don't see any benefit or advantage in other people review[ing] our administrative and health records.” Other potential participants were reluctant to provide a signature without the presence or advice from a trusted family member: “I am not signing any document without the presence of my son,” one female participant said, “because I do not understand many things and I am afraid to get into trouble.”

To address these issues, we revised the forms to be shorter and simply describe the project. Final versions of Spanish and Mayan informed-consent forms can be found at (Aguila et al., 2015). We also modified the informed-consent process to promote an exchange of information between the interviewer and respondent. Revised forms submitted to the RAND HSPC eliminated the requirement for the participant's signature, replacing it with the interviewer's signature as a “witness to informed consent.” That is, an interviewer's signature would attest that the interviewer had reviewed the content of the consent form with the respondent, offered appropriate answers to any question or concern of the potential participant, and obtained oral consent from the respondent to proceed with the survey. The forms allowing the field staff access to administrative or health records were held by government programs or health providers, and therefore required an actual signature from the participant. The revised versions of these forms allowed for a signature or fingerprint or even for a legally authorized representative to sign when the participant was unable to do so. In short, we simplified the consent forms for participation, while maintaining the basic elements of informed consent required by the HSPC.

We found that it was difficult for the interviewers to introduce the project to the eligible adults, or their relatives, and to clarify all questions regarding the consent form. We resolved these problems through more training as well as positive feedback and reinforcement of interviewer activities by field supervisors and project coordinators. In our debriefings, we discussed the field-test experience with interviewers and identified processes or procedures with which interviewers experienced difficulty and needed modification. In our field-test observations, we found that interviewers struggled with how to present the study to respondents, succinctly answer questions posed by respondents or their friends and family, and persuade respondents (or gatekeepers) to sign the consent forms and participate in the study.

**Figure 1:**
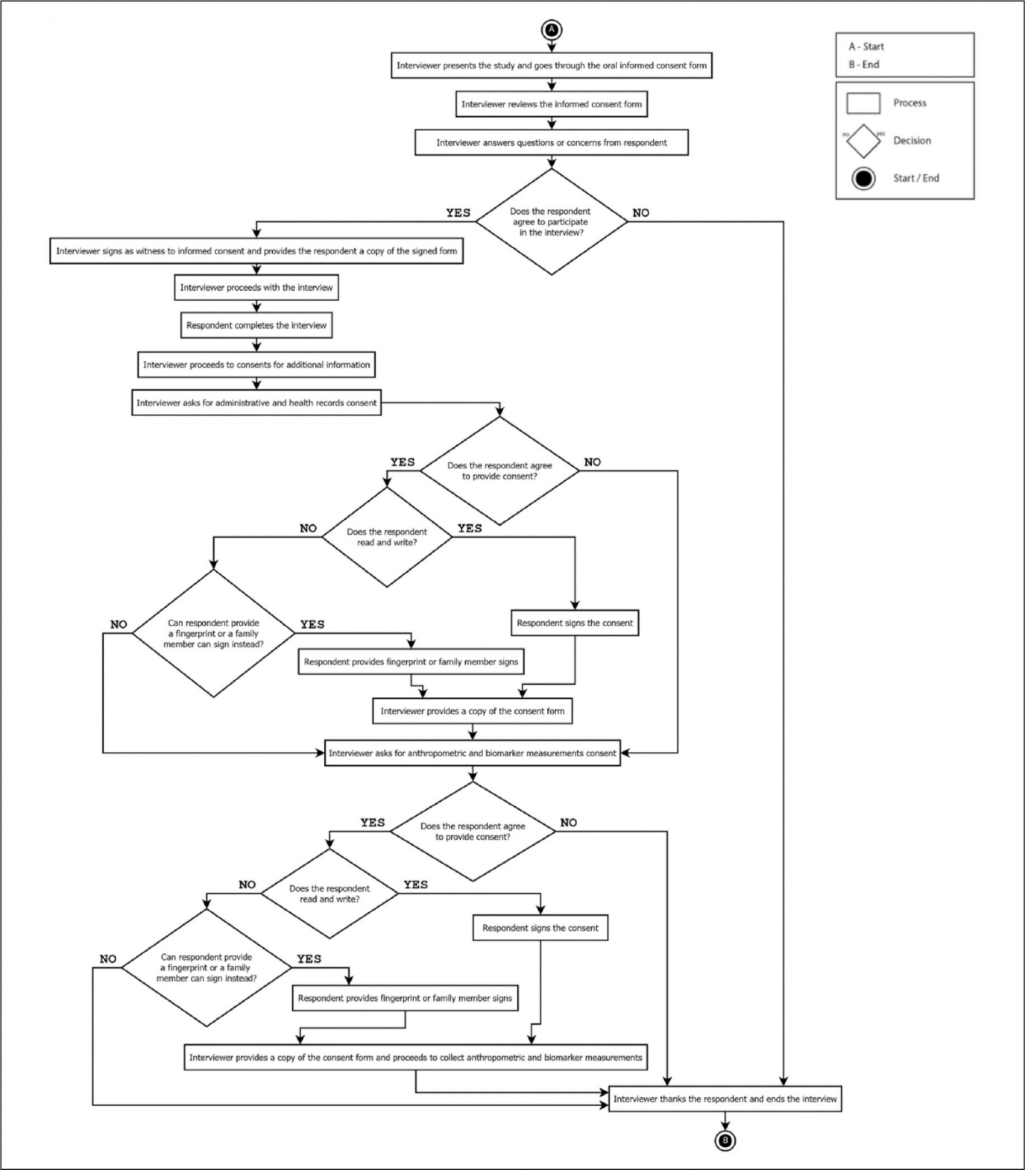
Interview and informed-consent process work flow. *Source.* Authors of this manuscript with information on the informed-consent process.

We instructed interviewers to ask for written informed consent for anthropometric and biomarker measurements after the individual interview, which only required oral consent, was finished. This allowed the interviewer to establish rapport with the respondent, and considerably improved the consent rate for more invasive procedures. Figure 1 shows the flow of the informed-consent process for the overall project.

Ultimately, the interviewers were able to obtain consent from a high proportion of eligible individuals. Table 1 summarizes the number of individuals in the two localities, Valladolid and Motul, where baseline data collection began in 2007. Overall, 99.8% of the eligible individuals gave their initial consent in the baseline survey, 94.5% did so for the first follow-up, and 91.8% did so for the second follow-up. Consent rates were higher for Mayan than for Spanish speakers across the three waves. The decrease in the proportion of eligible individuals who completed and gave their consent to participate in the follow-up survey was due to illness, death, refusal, or address change. There were no reports of eligible individuals declining to participate in the follow-up surveys because of issues related to the informed-consent form.

In Table 1, we can observe the proportion of individuals who, in Spanish or Mayan, gave written consent for access to their administrative and health records and for anthropometric and biomarker measurements. These written-consent rates were lower than the initial oral-consent rates to respond to the survey. These differences indicate that respondents were aware they could provide, or refuse, consent for different elements of the process.

## Discussion

As noted by Pappworth (1967) and Beecher (1966), the issue of obtaining informed consent among the marginalized populations deserves special attention. Dula (1997) similarly notes several reasons why implementation of research with adequate informed consent is especially relevant among ethnically and financially marginalized populations. These include the history of abuse of informed consent that may cause distrust, particularly among minority populations. Obtaining the consent of such individuals “calls for community consultation, education, and involvement.” These can be difficult to obtain among such populations.

Other studies including minorities have found that trust is key for survey participation. Barata et al. (2006) examined cross-cultural perspectives on informed-consent procedures in medical research among Portuguese Canadian and Black Caribbean Canadian immigrants. In both groups, the key to attaining informed consent was the degree of trust or mistrust arising in the process. The Portuguese considered interpersonal trust relevant to participation, while Black Caribbean respondents reported establishing trust to verify the legitimacy and safety of the study. The Black Caribbean respondents suffered more fear and anxiety about participating due to mistrust of the research and because the study could stigmatize their community. Even increasing participation in the focus-group session was difficult and only accomplished when a well-known community member asked for participation.

A number of scholars have paid increasing attention to informed-consent procedures for indigenous (Native American) populations. Ellis and Earley (2008) explored the value of employing culturally sensitive informed-consent procedures, such as offering *asemaa* (tobacco ties) symbolizing reciprocity among different cultural groups. They suggested that, in eliciting informed consent from an indigenous population, researchers ought to go beyond debriefing their research procedures and build on a more culturally sensitive researcher–subject relationship that embodies “the American Indian worldview involving collectivism, collaboration, compassion, and courage” (Ellis & Earley, 2008; Portman & Garrett, 2005). Informed consent is especially important for preventing exploitation of minority participants (Dula, 1997), but over-protection can lead to a complete exclusion of minorities from research (Elks, 1993; Smith, 2008).

Informed-consent processes on social research are less standardized than those in medical or clinical research. Some researchers (Beauchamp & Childress, 2012; Doyal, 1998) have argued that social-research ethics should follow medical- and clinical-research ethics emphasizing the values of autonomy of subjects, non-maleficence (“Research must not inflict harm”), beneficence (“Research should benefit others”), and justice (“People must be treated equally within the research process”; Wiles, Heath, Crow, & Charles, 2005). Social-science researchers (Goodwin, Pope, Mort, & Smith, 2003; Punch, 1998; Wiles et al., 2005), however, have criticized such a strict adherence to ethical principles as not reflecting the reality of social research where ethical dilemmas encountered are highly context-specific. Warnock (1998), for instance, contended that there could be cases, such as in conducting research with mentally incompetent participants, where these principles are breached. Tobias (1998) similarly suggested that social science demands much more lenient imposition of informed-consent requirements, given that informed consent for social research is often difficult to obtain. As Couture (2008) suggested, in social-science research the “benefits and costs of informed consent should be carefully considered in each situation (p. 1).”

The simplicity or complexity of developing adequate informed-consent procedures depends on the location of the project, specific cultural or demographic characteristics of the target population, the nature of the research proposed, requirements of participating institutions, and local rules and regulations on conducting research (Chin et al., 2011; Kripalani et al., 2008). For our Yucatan research, the lack of a LREC affected both researchers and respondents. For researchers, this lack made it more difficult to conduct collaborative binational research. Such difficulties reduce incentives to conduct research among marginalized communities, reinforcing their marginalization.

Adapting the informed-consent process to be more culturally competent required additional resources and staff training, and delayed implementation of the study. Doing so, however, allowed us to conduct successful research with a marginalized elderly population in a developing country without international IRB standards or procedures.

Following a dialectic approach, we conducted initial field-testing with extensive debriefing of the field staff about their experiences, and used this and local researcher expertise in primary data collection to restructure our interview forms. Our pilot test in Spanish helped us realize the importance of translating the informed-consent protocols and survey instruments to Mayan. One of our subsequent pilot tests completely focused on the Mayan-speaking populations, helping us adapt our process to their particular characteristics.

The project would not have succeeded without an iterative process of cultural exchange and adaptation that reflected true commitment to the ethical principle of respect for persons. One of the key contributors to the success of a research project of this type is interviewer training. We conducted comprehensive training sessions to instruct the interviewers about the importance of the informed-consent process. Training the project staff about the Mayan culture, traditions, and role of the elderly in society helped the research team understand the need to conduct research for this marginalized population. We collaborated with a local expert on Mayan culture who gave a lecture during field staff training.

The collaboration with our IRB was also key in the redesign of the informed-consent process. Harmonizing U.S. and Mexican regulations was always a focal point. It may be worth noting that, at RAND, PIs are regularly invited to HSPC meetings where their projects are reviewed. This allows for interaction and clarification between the researcher and the full IRB committee. While field-testing of instruments and processes can be challenging for projects with time and budget constraints, we found it to be vital in ensuring that our project was conducted with both ethical and methodological rigor.

We tried to ease participants’ fears of exploitation by allowing for oral or fingerprint consent. We implemented a staged process of consent. For the interview, we were able to rely on oral consent, with interviewers signing the consent form, indicating their role as “witness to informed consent.” This allowed respondents to feel at ease with interviewers. It also allowed interviewers to establish rapport with respondents before asking for a signed authorization to conduct more invasive tests, including anthropometry. When dealing with illiterate persons, interviewers were authorized to collect the signature of a family member on behalf of the older relative or a digital signature (i.e., a fingerprint) from the participant, with interviewers then also signing as “witness to informed consent.”

## Conclusion

In this article, we presented the experience of a group of researchers from a U.S.-based institution involved in research in a developing country, facing different challenges with the target population. These challenges included the target population's age, physical or mental frailty, reduced autonomy and privacy, low levels of schooling, and language challenges. In spite of these challenges, we were able to develop adequate consent forms, building on previous experience acquired in our own institution by other research groups facing similar conditions, and submitting our initial consent procedures, including informed-consent forms, to several iterations field-tested in subsequent pilot runs.

One of our most important lessons is that of involving local experts. Our project, though binational and cross-cultural from the outset, underwent even more cultural adaptation than anticipated. By approaching this project with a sense of mutual respect and flexibility, and by partnering with local researchers familiar with the nuances of indigenous norms, we were able to adapt our processes in ways that dramatically increased their acceptability to the target population. Specifically, we are grateful to a local researcher expert on primary data collection who participated in field-tests and debriefing sessions that shaped the informed-consent protocol. We intentionally chose project interviewers from the communities where they would be working, and who were able to recognize and explain the discomforts respondents had with the original consent forms and procedures. We accommodated participants’ cultural preferences by conducting interviews in their native language, with participation of the whole household.

Overall, there must be ongoing dialogue about whose authority should take primacy in decisions regarding “best practices” for informed consent when different stakeholders hold divergent viewpoints. U.S.-based IRB bodies and federal regulations may have quite different expectations for consent procedures than LREC. LRECs in turn might hold very different expectations than the target population has. Better understanding how to balance these competing demands and interests is critical as scientific inquiry becomes increasingly global. Future empirical research should show how research with vulnerable groups can align with the ethical imperatives that lie at the heart of research on human subjects: minimizing harm, maximizing benefit, respecting human dignity and autonomy, and striving to distribute equitably the benefits and burdens of research.

## Figures and Tables

**Table 1:** Number of Eligible Individuals and Individuals With Consent for Baseline, and First and Second Follow-Up and Interview Language.

	Eligible	General survey consent	Administrative and health records consent	Anthropometric and biomarker consent
Baseline	2,512	2,508 (99.8%)	2,484 (98.9%)	2,354 (93.7%)
Spanish	2,103	2,099 (99.8%)	2,079 (98.9%)	1,963 (93.3%)
Mayan	409	409 (100.0%)	405 (99.0%)	391 (95.6%)
First follow-up	2,278	2,152 (94.5%)	2,104 (92.4%)	2,068 (90.8%)
Spanish	1,874	1,765 (94.2%)	1,731 (92.4%)	1,697 (90.6%)
Mayan	404	387 (95.8%)	373 (92.3%)	371 (91.8%)
Second follow-up	1,959	1,799 (91.8%)	1,779 (90.8%)	1,653 (84.4%)
Spanish	1,547	1,395 (90.2%)	1,378 (89.1%)	1,263 (81.6%)
Mayan	412	404 (98.1%)	401 (97.3%)	390 (94.7%)

*Source.* Authors of this manuscript with information from the ENCAHEY (Survey of Household Economic Characteristics in the State of Yucatan or Encuesta de Características Socioeconómicas del Hogar en el Estado de Yucatán) surveys.

*Note.* The number of eligible individuals for the first and second follow-up does not include persons who died or could not be contacted. The difference between eligible and those who gave their consent is non-response due to illness, death, refusal, or address change.
